# Supplementary material for: Resuscitation simulation among people who are likely to witness opioid overdose: Experiences from the SOONER Trial
Source: PLoS One. 2022 Jul 5;17(7):e0270829. doi: 10.1371/journal.pone.0270829 (PMC9255733; doi:10.1371/journal.pone.0270829)
Supplement: S1 Text — (DOCX) [file pone.0270829.s001.docx]

**Orientation Guide**

Orient the Participant to the room, the equipment (including the manikin), the people involved.

**VIDEO TOUR**

- For this simulation, you will be entering into an apartment. This video tour is to help you know what to expect when you enter the room.
- You will notice a wall divider to your left. The space behind the wall is not part of the simulation – please do not go back here
- When you enter, you will see that you are in a living room – there is a couch, table, television, (MAKE A LIST OF ALL ITEMS ONCE FINALIZED)
- On the desk, you will find a telephone. If you need to make a call during the simulation, please use this phone and not your own cell phone. Use this phone to make any calls that you would make in real life. Don’t worry – it will connect you directly to me.
- There are no surprises or anything hidden in the apartment. Please act as you normally would when visiting a friends home.
- If you have any questions, please ask one of the research staff now.

**MANIKIN – NASAL SPRAY**

- For the simulation, you will be interacting with a mankind. It may seem strange as it is not a real person, but please speak, touch and behave as though it’s a normal person.  Treat them as your friend and act as you would in real life.
- There are a few features of the manikin we want to point out to you – if the person is breathing – you will see the chest rise and fall, and hear breath sounds – (SHOW THIS)
- The manikin is heavy, please be careful not to hurt yourself if you would like to move it.
- You can put things into his nose or mouth if needed
- The more you behave as though this is a real person, the more successful the scenario can be. Please feel free to ask any questions you may have about the manikin now.

**MANIKIN - INJECTION**

- For the simulation, you will be interacting with a manikin. It may seem strange as it is not a real person, but please speak, touch and behave as though it’s a normal person.  Treat them as your friend and act as you would in real life.
- There are a few features of the manikin we want to point out to you – if the person is breathing – you will see the chest rise and fall, and hear breath sounds – as shown here
- The manikin is heavy, please be careful not to hurt yourself if you would like to move it.
- If you would like to inject the manikin with medication, you can do so on the pads on his upper arms or thighs (SHOW AREAS)
- The more you behave as though this is a real person, the more successful the scenario can be. Please feel free to ask any questions you may have about the manikin now.

**Orientation script:**

*Thank you for coming in to complete the follow-up assessment. We’d like you to take part in a simulated scenario – this will be a role-play in which you will demonstrate the skills you learned from the training to show us what you would do if you happen to be at the scene of an overdose. I will show you the simulated apartment space first. I will tell you about the scenario and answer any questions that you have after the paperwork.*

*We have set up the simulation in another room. I will take you there soon. Before we begin, I want to describe what you should expect and to answer any questions you have. Remember this simulation is not meant to judge you as a person, do the best you can. We hope that this simulation will be a safe place where you can try things to help out a friend, no harm will come to him or you, try your best. This is the perfect time to try things, mistakes are totally allowed! At the end we can give you some feedback and talk about what worked well and what didn’t. You can also tell us what worked and what didn’t work well – we will learn from you. Which naloxone kit were you trained with? We have intramuscular, and two different intranasal kits for you to choose from. Please use the kit that we provide you, not your own.*

*Injection:*

*Walking into the simulation room, you will see a life-size medical manikin dressed in street clothes– we call him Joe. It might look a little strange but it’s the best we can do.* ***There is also some drug paraphernalia*** *to set the scene and a number of things you might choose to use to respond to the overdose– what you use will be up to you. Treat him as best you can as if Joe is a friend or someone else who might be overdosing. The scenario will be video-recorded by a camera– only the members of the research team will have access to this video. We will also record the debrief so that we can learn what worked well and what could have gone better. In the other room there will be one of us watching, we are here for the research part of this project, not to judge you!*

*Ingestion:*

*Walking into the simulation room, you will see a life-size medical manikin dressed in street clothes– we call him Joe. It might look a little strange but it’s the best we can do.* ***There are also some pills*** *to set the scene and a number of things you might choose to use to respond to the overdose– what you use will be up to you. Treat him as best you can as if Joe is a friend or someone else who might be overdosing. The scenario will be video-recorded by a camera– only the members of the research team will have access to this video. We will also record the debrief so that we can learn what worked well and what could have gone better. In the other room there will be one of us watching, we are here for the research part of this project, not to judge you!*

*All:*

*Although we want this to be like a real-life scenario. You can also tell me if there is anything you would like an available bystander to do, but I will not be physically involved in the scenario; you will be the only one directly interacting with Joe.*

*After you walk into the simulation room, I will start the camera to the simulation will begin. There is no set time for the scenario– take the time that you need to complete the necessary overdose response steps, but remember that in an actual overdose, every minute counts. Act as you would in real life. I will let you know when the simulation has ended.*

[Walk into simulation room and orient to objects in the room.]

*Remember: If the scenario makes you uncomfortable, you can stop at any time. Just say “Stop the simulation” and we will stop the scenario. At the end of the scenario, we will have a chance to discuss how the scenario went for you, including what you did well and how you can improve. Do you have any questions before we begin?*

*Injection:*

*Its 6 pm on a Saturday night, you are going to your friend’s Joe’s apartment after work.* *You know Joe is there waiting for you****, and that he sometimes uses alone.*** *You have known Joe for the last 3 months.*

*Ingestion:*

*Its 6 pm on a Saturday night****,*** *you are going to your friend Joe's apartment after work. You know Joe is there waiting for you****, and that he was recently been prescribed [insert drug here] because of his [insert diagnosis here] diagnosis.*** *You have known Joe for the last 3 months.*

*All:*

*Any questions? Are you ready? Remember treat him to the best of your abilities as if Joe is a friend or someone else who might overdose. If you have witnessed or responded to an overdose, you may find this simulation difficult or triggering- remember, you can stop the simulation at any time by telling us to stop.*

Once the participant has been oriented to the room, the research team member and participant will go back to the anteroom to answer any last questions and then deliver the stem.

- 1. Scenario

1. Turn on GoPro, confirm image view / angle and start recording.
2. Observe participant.
3. After naloxone administration or if no naloxone administration within 5 minutes, turn on the audio track of ambulance sirens. Sound effects of the ambulance siren will get louder and louder suggesting that police or EMS is on their way.
4. Simulation is complete at EMS arrival after 8 minutes or if the participant indicates that they have completed their resuscitative actions, or participants takes no further action, stop the simulation by entering the room.

*Thank you for participating in the simulation. We are now done the scenario. Congrats! Take a deep breath and lets move over to the couch/chairs and discuss. If you would like, we can take a moment to review what you did.* [if participant agrees, proceed to debrief]
