# Supplementary material for: Resuscitation simulation among people who are likely to witness opioid overdose: Experiences from the SOONER Trial
Source: PLoS One. 2022 Jul 5;17(7):e0270829. doi: 10.1371/journal.pone.0270829 (PMC9255733; doi:10.1371/journal.pone.0270829)
Supplement: S2 Text — (DOCX) [file pone.0270829.s002.docx]

**Debriefing (PEARLS Debriefing Framework)**

1. Offer snack.
2. Debrief Script:

- Reaction*:“How did that feel?”*
- Description: *“Can you summarize briefly what the sim was about? What were the main issues you had to deal with?”*

1. Analysis: Debriefer to pick one or more of the three methods below:
   1. **Participant Self-Assessment (e.g. Plus-Delta)**

- *“What aspects of the case do you think you managed well?”*
- *“What aspects of the case would want to change, or try again?”*
  1. **Directive feedback and teaching**
- *“I noticed you [insert performance gap here]; Next time, you may want to … [close gap]…because [provide rationale”*
  1. **Focused Facilitation (e.g. Advocacy-Inquiry)**
- *“I noticed you [insert action here], was there a reason you chose to do that?”*

1. Application/Summarizing:

- “*Are there any outstanding issues we haven’t discussed yet before we start to close?***”**
- *“Is there anything you would like to try again?”*
- *“Let’s review the 5-steps approach to respond to an opioid overdose….[show steps]”*

1. Learner Driven: *“I like to close the debriefing by having tell me one or two take-aways that will help you in the future.Do you want the opportunity to practice any of these skills on the manikin?”*

*Refer participants to SMH community CPR course if interested.*
