# Supplementary material for: Resuscitation simulation among people who are likely to witness opioid overdose: Experiences from the SOONER Trial
Source: PLoS One. 2022 Jul 5;17(7):e0270829. doi: 10.1371/journal.pone.0270829 (PMC9255733; doi:10.1371/journal.pone.0270829)
Supplement: S3 Text — (DOCX) [file pone.0270829.s003.docx]

**Qualitative Interview Guide**

Follow-up Interview Guide with Trainee Participants

N.B. These questions serve as a guide only. It will be administered by researchers who will be able to probe and discuss topics as they arise with participants.

The audio files will be transcribed by an external transcription service following the interview and the recordings (audio recording and electronically saved audio file) will be destroyed following transcription.

During the participant follow-up interview no personally identifying information will be requested. Any personally identifying information voluntarily given during the interviews will be deleted from the final transcripts.

To avoid over recruitment and excessive qualitative data in the feasibility phase, the qualitative interviews will be stopped after approximately 25 interviews.

Preamble:

*Thank you so much for taking the time to speak with me today. I’m a researcher here at St. Michael’s and part of the SOONER study team. The purpose of the interview is to get your impressions of what it was like to take part in the study, any suggestions you may have for us for improving the study, as well as to ask you to share some of your/family’s/friend’s experiences of taking/using opioids and with using naloxone.*

*You can skip any question that you wish, and you may stop the interview at any time. If you’d like to take a short break, please let me know and we can do that. No identifying information about you will be shared with anyone outside the study team. Your care at St. Michael’s Hospital will not be affected, regardless of whether you choose to participate in the interview or not. We want to keep this anonymous, so during the interview, please try and avoid using your name, or the names of other people such as friends or family members. Any identifying information will be removed from the written transcription and from reports. We may use a quote from your interview in a publication, however your identity will not be revealed. All the information provided by you will be kept strictly confidential and we hope you will feel able to speak freely as we genuinely want to hear your perspective. Also, there are no ‘right’ answers to any of my questions – it’s your views that are important.*

*Before we start the interview, can I just check you are comfortable with the interview being recorded? Remember, you may stop the interview or ask questions at any point. Do you have any questions or concerns before we start? If not, let’s begin:*

**Section A: About you and your experiences of participating in the SOONER study**

1. I’m going to start by asking you to tell me a little bit about yourself. We haven’t met before as part of the study, so it helps me to get to know a bit more about you.

*Potential probes:*

- 1. *What part of the city are you currently living in? Was it hard to get here for the interview?*
  2. *Do you live with anyone? Tell me about that.*

1. Tell me a little bit about your health. How would you describe it? Do you have any health concerns? What are they?
2. What prompted you to volunteer to participate in the SOONER study?

*Potential probes:*

1. *Have you ever participated in a similar type of study? If so, what was that like?*
2. *How did you first hear about the study? Who told you about it?*
3. *What interested you about the study?*

**Section B: Suggestions for improving participants’ experiences in the SOONER study**

1. Thinking back to how you heard about the study, is there anything that would have been helpful in terms of how you were first informed about the study (e.g. flyers posted in clinic, invitation by card, how clinicians/hospital staff approached you, knowing that there are two parts to the study, etc.?)
2. What about how study staff approached you? What were your interactions with them like when you first met them? Was there anything they could have done better at the beginning?
3. What about when you came back to the hospital to do the simulation and feedback session? Was there anything about that that you didn’t like? If so, what was it?

*Potential Probes:*

- 1. *How easy was it to find the simulation centre?*
  2. *How comfortable did you feel in the simulation centre?*
     1. *How could we make you feel more comfortable?*
  3. *Was there something you liked about it? What was that?*
  4. *Was it easy or challenging to imagine that the simulation was ‘real life’?*
     1. *How could we make it feel more realistic?*
  5. *How did you feel about how you were treated by the study staff during this session?*
  6. *What were your expectations for the simulation? Did your experience match those expectations?*
  7. *Were the objectives and instructions for the simulation explained to you clearly by the study staff?*
  8. *Thinking about what resources/tools or things that were available in the simulation to help you, did you use anything in the room? Did you use anything that you brought with you? What was helpful /not helpful about those items?*

1. What was it like when study staff called you back, either with phone call reminders, or to do the final round of follow-up testing? Is there anything the y could have done differently? Anything they could have improved?

*Potential Probe:*

- 1. *How did you feel you were treated by the staff during these calls?*

1. Now that you’ve had the training, do you believe that you could use naloxone to help another person if you needed to? Tell me about that.

**Section C: Suggestions to improving the SOONER kit. Ask participants randomized to intervention arm only.**

1. Tell me about the SOONER kit and training. How did you feel about doing the training? How can we improve? What would you recommend?
   1. What did you think about the medium (animated video)? How about the length of the training?
   2. What did you think of images, colours, and or characters that were used?
   3. What do you think about the animation in terms of tone, clarity, brevity, memorability?
   4. What did you think of the voice over or music?
   5. Anything else that stood out to you? – good or bad
   6. How does it compare to any other leaflets/posters or other things that are on drug use?
   7. How does it complete to any other leaflets/poster or other things that are on first aid?
   8. How do you feel about the kit, its size and colour, what it is made of, how it’s organized?
   9. How have you been carrying it/stowing it?

**Section D: Experiences of taking an opioid and with using naloxone**

1. Now I’d like you to think back to when you or your family or friend first began taking an opioid such as fentanyl, morphine, dilaudid, heroin. Can you remember what the circumstances were when you/they started taking it/them? Tell me about that. Was it prescribed or not?
2. What has been your or your family or friend’s experience of taking opioids since that time?
3. Who, if anyone, knows you or your family or friend take opioids? How did they find out? Who do you tell? Under what circumstances? Tell me what that is like for you.
4. Now I’d like to ask you about your experiences with the naloxone you were given since starting the study. Have you had to use the naloxone? If so, what have been your experiences with using it in these past few weeks?
   1. Had you ever heard of naloxone before taking part in this study? Had you ever taken naloxone before being part of the study? Tell me about that.
   2. Have you ever administered naloxone before?
   3. Had you heard of naloxone being given in a nasal spray before this study? Tell me about that.
   4. Do you carry the naloxone you were given in the study with you? What is that like? How do you feel about it?
   5. When do you carry it? How do you carry it?
   6. How many do you carry with you?
   7. Did you show it to anyone?
   8. Do you let other people know that you’re carrying it? Who do you tell? Tell me about that.
   9. If you do let them know, how have they reacted? Have their interactions with you changed in any way, once they know? Tell me about that.
5. Now I’m going to ask you something I’m asking everyone in the study, don’t think too much about it, just say whatever pops into your head. List any words that pop into your head when I say the term ‘opioid’. ()
6. And now I’m going to ask you to do the same thing, but with the term ‘naloxone’ and then the people in the training.
7. Do you feel that people who are taking opioids face any barriers to healthcare here in Toronto? Tell me about that.
8. Tell me about the SOONER kit and training. How did you feel about the video? About the kit? How can we improve? What would you recommend?
9. Do you feel that there are any barriers to naloxone distribution (getting naloxone to people who might need it and the people that care about them)? Tell me about that.

**Section E: Closing out the interview**

1. That’s all the questions that I had for you today. But before we finish is there anything else that you would like to add? Are there any other topics you would like to discuss that I haven’t covered about the study or naloxone?

Thank you so much for taking the time to speak with me today!
