# Supplementary material for: Resuscitation simulation among people who are likely to witness opioid overdose: Experiences from the SOONER Trial
Source: PLoS One. 2022 Jul 5;17(7):e0270829. doi: 10.1371/journal.pone.0270829 (PMC9255733; doi:10.1371/journal.pone.0270829)
Supplement: Attachment — Submitted filename: Response to Reviewers.docx [file pone.0270829.s004.docx]

**Response to Reviewers**

Thank you both for this thoughtful commentary and insights on how to improve this paper. Please find our responses to the reviewers’ suggestions below:

REVIEWER 1
***I am confused. I understand it is a part of SOONER Trial but what was the aim of that paper. The simulation protocol is unclear, especially in part simulation scenario. What was the learning objective in that simulation scenario?***

To improve the clarity of the study design we made the following changes:

- Introduction: we revised the description of the SOONER study to be more concise and clearer. The last two paragraph of the introduction provide a better description of the learning objective of the initial SOONER study and the current study. We removed the description of the study objective as being two-fold to simplify the study goals. The primary objective is to describe the experience of participants. This necessitates a description of the simulation protocol.
- Learning objective of the simulation scenario (see intro): “*The primary objective of the SOONER study was to evaluate whether a novel point-of-care opioid overdose education kit could lead to improved resuscitation outcomes compared to the current standard of care which involves a referral to a local pharmacy or naloxone distribution site (18) …* T*he ability of participants to successfully resuscitate the mannikin (administer naloxone, perform CPR, etc.) was evaluated and their performance during simulation was used as the primary outcome measure for the SOONER study (18).” (Page 4, Line 97)*
- Methods: removed the description of the overview and scope of the SOONER project, this is not directly relevant to the present study and can be accessed through the references which are provided in the text.

***In results we don't have any results of the scenario activity. We don't know in what percentage naloxone was administrated and if CPR was performed. If yes what was the activity timeline. When naloxone was administrated, when 911 call was performed? What kind of device/protocol was used for quality of CPR judgment? Chest compression fraction - CCF? In fact we don know if CPR was necessary in scenario and if any of participants performed it before emergency arrival. So in fact the title of manuscript should be rephrased. Resuscitation is the first word in the title but we don't know if resuscitation was the learning objective or was performed in any case?(!) Or maybe I am wrong. if yes it should be indicated what is the aim of paper study - on fact the results include only debriefing part.***

The goal of our study is to describe and understand the personal experience of participants with the simulation process. A description of the performance of participants on the simulate overdose or of the outcome measures is the subject of the larger SOONER trial and outside the scope of this study. We have amended the introduction to clarify this:

*“The purpose of this study is to describe* *the experience of participants with the simulation process in the SOONER study. The objective performance of participants during the simulated overdose represents the outcome measure of the SOONER study and will not be described in this paper. Rather, we seek to understand the perspectives and the subjective experience of participants with the simulation process.”* (*Page 4, line 103)*

We also provided a description of exactly what participants were required to do during the simulation protocol:

*“To achieve a successful resuscitation, participants were required to perform what they had been taught during their educational intervention. At a minimum, participants were required to administer intranasal naloxone and perform chest compressions on the manikin, but their ability to recognize the emergency, position the manikin, activate Emergency Medical Services (EMS), and organize their approach was also evaluated. The overdose simulation was recorded, and the ability of participants to navigate through the resuscitation algorithm and complete each of its steps was evaluated independently by two clinicians blinded to allocation” (Page 7, Line 161)*

***The participants demographics and table 1 should be included in methods part. The participants profile is not the result of the presented study!***

We changed the location of the Demographic Table in Methods as recommended.

**REVIEWER 2**

***I think that the qualitative design provides a lot of information, however, a mixed design with validated instruments useful in simulation would have helped to recruit more evidence to the scientific community. There are instruments such as Student Satisfaction and Self-Confidence in Learning Scale” that assesses the level of satisfaction and self-confidence of the participants in a simulation activity or the “Debriefing experience scale” that offers information about the debriefing experience. I suggest that in later studies a mixed design can be considered.***

The Student Satisfaction and Self-Confidence in Learning Scale has been developed and validated in health care professional simulation participants, and especially nursing students. To our knowledge, it has not been validated among lay responders, non-healthcare simulation participants, or among people who face structural marginalization such as people who use drugs. Future work could involve the development and validation of an appropriate tool for lay providers or people responding to overdose emergencies, but we do not feel that applying the Student Satisfaction and Self-Confidence in Learning Scale would add validity to our study. We are not aware of a well-validated or culturally appropriate quantitative tool for the population and circumstances of our study, and believe therefore that qualitative inquiry and interview-based data was the most appropriate form of analysis.

***Regarding the simulation protocol used. I understand that the SOONER study was standardized, but I think that using an actor instead of a mannequin would have helped the participants to experience the situation more realistically. However, I see from the results that this was not a limitation for them. Likewise, using simulation strategies by repetition, such as the rapid cycle, I think would have helped the participants to increase their learning curve regarding resuscitation skills in the event of cardiorespiratory arrest due to overdose.***

It is true that the addition of a simulated standardized actor may have added value to their experience. The goal of our study was to understand specifically how participants experience simulation with a manikin as we believe that this form of simulation is generalizable to many other settings including lower resourced setting. This finding also supports a growing body of literature which suggests that the degree of fidelity may not be as important to promoting learning during simulation as previous believed. Further studies (building on our work of using simulation for patients at risk of an overdose) may incorporate actors or standardized confederates in order to explore their value.

Evaluating participant learning was outside of the scope of this study as we chose to focus specifically on the subjective experience of participants with the simulation process. We added a line to make this clearer in the introduction:

*“The purpose of this study is to describe the experience of participants with the simulation process in the SOONER study. The objective performance of participants during the simulated overdose represents the outcome measure of the SOONER study and will not be described in this paper. Rather, we seek to understand the perspectives and the subjective experience of participants with the simulation process.”* (*Page 4, line 103)*

We will consider integrating simulation strategies such as standardized simulated patients, and the rapid cycle in future designs that might seek to evaluate participant learning.

Regarding the specific recommendation to use an actor rather than a mannequin, we believe that an actor would be appropriate for a simulation scenario that was less reliant on peri-arrest and cardiac arrest physiology. Actors would be suitable for simulating decreased levels of consciousness, stupor, and other interactions, however, they are less well suited to simulate pulselessness, respiratory arrest, or allow simulation participants to perform invasive or painful interventions including intranasal medication administration, CPR, and mouth-to-mouth resuscitation. Actors are also not well suited to simulate death. Mannequins offer other deficiencies, for example, they are cold and cannot rouse realistically. In exploring and weighing these pros and cons of actors and mannequins, we chose to simulate this scenario with a mannequin.

***To understand how the post-simulation reflections were carried out, it is important to describe the debriefing process and what methodology was used. I suggest that it can be added to the manuscript***

Thank you for flagging this deficiency. We added additional clarification under “Data Collection” to address this:

“*The first source of data was the debriefing which occurred immediately following the simulated overdose and was guided by the Promoting Excellent and Reflecting Learning in Simulation (PEARLS) framework.*” *(Page 8, Line 181)*

We added the interview guide for both the PEARLS debriefing and the qualitative interview to the appendix section of this submission and have referenced this in the text.

*“The video recorded debriefing was led by the evaluating clinicians and lasted approximately 5-10 minutes (protocol in S2 Text). The second source was a more in-depth qualitative interview which occurred following the debriefing and was administered by researchers involved with the SOONER trial (protocol in S3 Text).” (Page 8, Line 185)*

***Regarding ethical considerations, it is not specified how the participants were informed, if they signed an informed consent and how said data was treated. I think it is important, due to the profile of the participant, to specify these data for the publication of this manuscript.***

Details about participant consent have been added to the ethics portion of the manuscript:

“*A systematic review on strategies to improve informed consent processes found that having a one-to-one discussion was the most effective way of improving participant understanding of the research process. Verbal consent was therefore obtained from participants in a setting that afforded ample time for discussion and questions. Study coordinators then documented that they obtained verbal consent on the paper consent*.” *(Page 9, Line 211)*

A line was edited in the methods to clarify how the data was treated: “*data from the interviews were coded and stored in a secure study database*”. *(Page 9, Line 192)*
